# Dispensing hope: leveraging distribution boxes to enhance low-barrier access to naloxone in healthcare settings

**DOI:** 10.1186/s12954-025-01229-5

**Published:** 2025-05-22

**Authors:** Nycole Kothe, Angela Gray, Sarah Guthrie, Michael Londner

**Affiliations:** WVU Medicine Berkeley Medical Center, 2500 Hospital Drive, Martinsburg, WV 25401 USA

**Keywords:** Naloxone, Distribution, Opioid, Substance use disorder, Overdose, Harm reduction

## Abstract

**Background:**

West Virginia, often regarded as the epicenter of the opioid epidemic, has consistently recorded the nation’s highest rates of opioid overdose deaths. The Eastern Panhandle, which includes Berkeley, Morgan, and Jefferson counties, mirrors this trend, with Berkeley County’s overdose death rate in 2020 exceeding triple the national average. Recent data, however, indicates a promising 37% decrease in overdose deaths in Berkeley County over a 12-month period ending August 2024, and a statewide decrease of 27.9%. This decline is suspected to be attributed to enhanced access to naloxone and medications for opioid use disorder, supported by community collaboration. Despite these gains, barriers persist for at-risk populations, necessitating strategies such as over-the-counter naloxone availability.

**Methods:**

The implementation of naloxone distribution boxes in healthcare settings, initiated in Berkeley Medical Center’s emergency department, marks a significant advancement. These boxes, accessible at all times and stocked with OTC (over the counter) naloxone kits, were placed in visible areas of the building vestibule to increase community access to naloxone. Results: Over a six-month period, these boxes distributed 2,383 naloxone kits, significantly surpassing physician-ordered distributions of 17 kits over the same period. Conclusion: Distribution boxes allowed for delivery of large quantities of naloxone compared to the physician-ordered distributions. Future efforts aim to expand naloxone availability in community settings to sustain and further reduce overdose fatalities.

## Background

Often referred to as “ground zero” of the opioid epidemic, West Virginia has consistently recorded the highest opioid overdose death rate in the nation. The Eastern Panhandle of West Virginia, which includes Berkeley, Morgan, and Jefferson Counties, has historically followed the state trend; in 2020, Berkeley County had a rate of 94 fatal drug overdoses per 100,000, more than triple the national average of 29.05 per 100,000 in the same year [1, 2]. However, recent data shows encouraging results which indicate that overdose deaths in Berkeley County, WV decreased by 25% over a 12-month period ending in September 2023, while the state rate increased over the same period [3, 4]. Current data shows an even greater 37% decrease in overdose deaths in Berkeley County in a 12-month period ending in August 2024 and a 29.2% decrease in Jefferson County [3]. Over the same time, there was a 27.9% decrease in overdose deaths in West Virginia and a 21.7% decrease nationally [4].

Berkeley County is located in the Eastern Panhandle of West Virginia and has a population of 136,287 [5]. Berkeley County is in a tri-state area with Virginia and Maryland which puts it near major urban centers, such as Baltimore and Washington DC, increasing exposure to novel and often deadly drug supplies. The county also runs alongside Interstate-81 which is colloquially known as the “heroin highway” due to its association with drug trafficking. Berkeley County is served by Berkeley Medical Center (BMC) as well as the Martinsburg Veterans Affairs Medical Center. BMC Emergency Department is the busiest in the state, averaging more than 56,000 patient encounters annually [6]. Jefferson County, which has a population of 61,264, and the surrounding areas are served by Jefferson Medical Center, a 25-bed critical access hospital [7]. In 2023, Berkeley County had 452 reported emergency room visits related to overdose, averaging approximately 38 visits per month. In 2024, that number decreased to 378, or about 32 visits per month. Jefferson County reported 176 overdose-related ER visits in 2023, an average of nearly 15 per month, and 104 in 2024, averaging about 9 per month [8].

The reductions in overdose fatalities are encouraging and raise questions to what is driving the decrease. There is no indication that the drug supply has become less potent during this time. In fact, the opposite is likely true due to widespread use of fentanyl, synthetic opioids, and xylazine which can increase the risk of fatal overdose [3, 4, 9]. Local epidemiologists and health experts speculate that this decline may be attributed to increased access to the overdose reversal medication naloxone, as well as medications for opioid use disorder (MOUD) and widespread community collaboration.

Starting in *2022*, efforts were made to revitalize the local tri-county coalition, West Virginia East Coalition for Addiction Recovery and Education (WE CARE), and establish the Virginia West Virginia Maryland Tri-State Collaborative, allowing key community stakeholders including healthcare, schools, and law enforcement to work together to address challenges related to substance use disorder and better coordinate care and resources. Strong community collaboration led Berkeley County law enforcement officers to carry naloxone over the past three years, using it to protect both police canines and members of the public. Additionally, Berkeley County Emergency Medical Services (EMS) have been leaving naloxone behind when responding to overdoses for the past year. A harm reduction program is available in Berkeley County, offering both syringe access and naloxone. Community members can also access naloxone through the WVU CORE Team and local Quick Response Team (QRT), whose mobile units have offered resources and responded to overdoses in the community for several years.

BMC ED began initiating treatment for opioid use disorder in 2016 with the start of the Bridge to Recovery Program. The “Bridge” program offers MOUD induction (buprenorphine) and an ongoing prescription to last until the patient’s follow-up appointment with an outpatient treatment facility. Local treatment facilities have limited capacity, often resulting in follow-up appointments scheduled several weeks after the patient’s ED visit. Since the start of the Bridge program, we have developed a comprehensive addictions program rooted in best practice, including a team of Peer Recovery Support Specialists (PRSS), often referred to as peers, that began providing services in BMC and JMC in 2022. Additionally, BMC offers SBIRT (universal screening, brief intervention, and referral to treatment), and harm reduction measures such as fentanyl and xylazine test strips, wound care education, and HIV, hepatitis, and syphilis testing [10]. Addiction services are primarily centered in the ED but have expanded to all inpatient floors through Project Engage, an initiative with grant support from West Virginia Department of Health and Human Resources, Bureau for Behavioral Health (BBH). The addition of peers allowed BMC and JMC to vastly increase the number of patients receiving services for substance use disorder (SUD) and referred to treatment. BMC Addiction Services completes an average of 32 Bridge appointments per month (September 2022–May 2024) and provides peer recovery coaching to approximately 82 unique patients per month (July 2023–June 2024). JMC has approximately 60 peer consults per month on average.

Although overdose fatality rates have shown recent improvement, they remain at epidemic proportions and continue to pose a significant public health threat, indicating a need for expanded access to naloxone for effective overdose reversal. In West Virginia, the State Health Officer has issued a statewide standing order that permits eligible organizations, including hospitals, to distribute naloxone without individual prescriptions. This standing order facilitates the dispensing of naloxone directly to patients in emergency departments and allows for the stocking of naloxone kits in publicly accessible distribution boxes [11]. To further improve access, the United States Food and Drug Administration (FDA) approved the first over-the-counter (OTC) naloxone product in March of 2023 [12]. However, barriers exist for high-risk populations to access the medication via this route. Pharmacies may be stigmatizing for patients with SUD, and medication costs (around $45 for OTC naloxone) may hinder access [13, 14].

The emergency department (ED) is the primary location where people who use drugs access medical care, making it an ideal setting to distribute naloxone [15]. Prescribing by physicians is not sufficient, however, with studies showing prescription fill rates as low as 2% [16]. A large-scale study revealed that ED patients treated for nonfatal overdose face high short- and long-term mortality rates, with 5.5% dying within a year—comparable to nonfatal myocardial infarction rates—and most deaths within the first month occur within 48 h of ED discharge [17]. Studies also show that take-home naloxone programs, where high-risk patients are sent home with medication in hand, significantly reduce mortality rates [18, 19].

In February 2022, BMC ED implemented its take-home naloxone program to provide patients with the medication before they leave the hospital. The take-home naloxone program gave providers the option to order naloxone for high-risk patients using “To-Go Meds” within the electronic medical record (EMR) system. Before dispensing via the Omnicell (an automated medication dispensing system), Addiction Services staff, including peers, provided training and instruction to patients on recognizing an overdose and administering the medication.

Though the take-home naloxone program was initially a success, distribution rates remained low. Addiction Services staff first attempted to increase distribution through verbal promotion of the program and conversations with ED physicians. During Addiction Services meetings and consultations with the California Bridge Program, extensive discussions focused on the concept of “naloxone vending machines” to enhance the distribution program [20]. The idea eventually shifted to newspaper-style naloxone distribution boxes, chosen for their simplicity, accessibility, and minimal maintenance needs. Here, we outline the results, successes, and barriers of the naloxone distribution box program over the first six months of implementation (December 2023-May 2024). This project offers a novel, low-barrier model for naloxone distribution through publicly accessible boxes, addressing persistent challenges in take-home naloxone uptake and potentially expanding the evidence base for harm reduction strategies in clinical and community settings.

## Methods

Securing buy-in from hospital leadership and support from community partners proved invaluable to launching a successful take-home naloxone program, an area where many institutions face challenges. In this case, leadership engagement was fostered through a collaborative, step-by-step approach that emphasized shared goals and patient safety. The first step was to establish take-home naloxone within the emergency department, via the “To-Go Meds” physician ordered system. A few passionate ED physicians took on advocacy roles, serving as “champions” for the program and helping to build internal momentum. Collaborative meetings with hospital pharmacists addressed operational details such as billing and storage, while efforts were made to communicate the program’s value and lifesaving potential. A key strategy for the addition of the distribution boxes included presenting a visual of the box along with funding information to senior leaders, helping to illustrate both their practicality and potential impact. Broad community support through coalitions further reinforced the program’s importance, helping to build a foundation of trust and shared purpose across the organization.

**Fig. 1:**
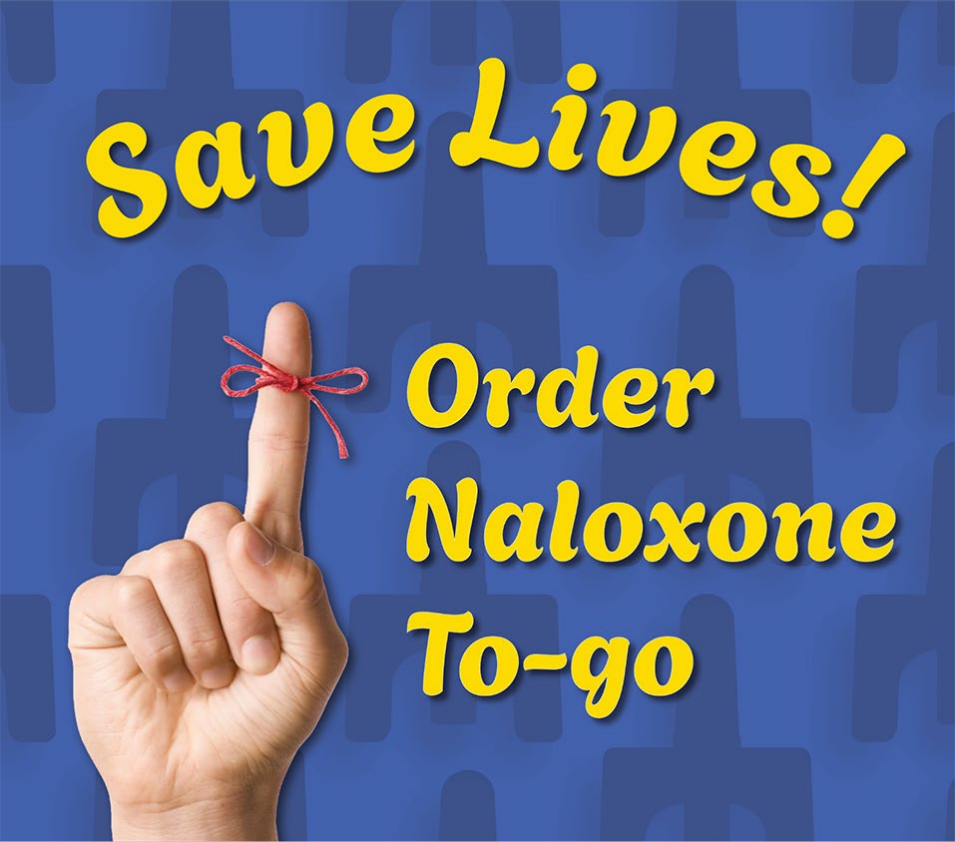
Image of take-home naloxone promotional mousepad design

On December 1, 2023, coinciding with the launch of the naloxone distribution boxes, take-home naloxone mousepads designed in collaboration with the California Bridge Program were placed at all emergency department computers. These mousepads were intended to remind physicians about the option to order naloxone in the EMR using “To Go Meds” and boost distribution (Fig. 1).

**Fig. 2:**
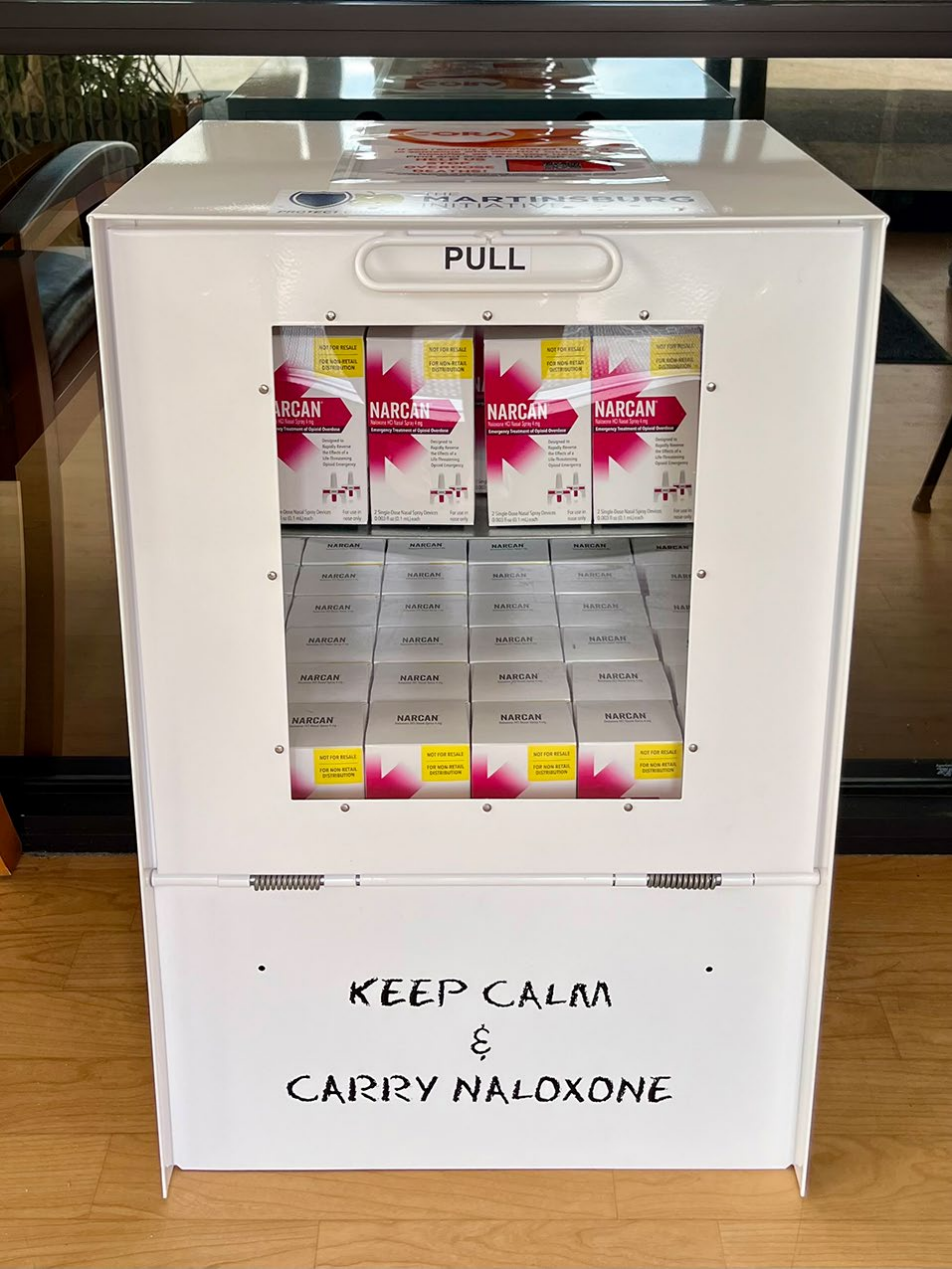
Image of naloxone distribution box placed in emergency department vestibule

Naloxone distribution boxes were ordered in phases through donations from community partners including the Berkeley County Commission, through the Day Report Center, as well as The Martinsburg Initiative. WVU Medicine Eastern Division’s Addiction Services leadership team worked with the University of Charleston to obtain the required OTC Narcan supply which is funded by grants provided to the state of West Virginia. The take-home naloxone program that relies on physician orders uses a non-OTC supply (Fig. 2).

The first distribution box was placed in the BMC ED entrance in on November 29, 2023. The box was placed in a visible location within the building vestibule to shield it from extreme temperatures and weather conditions, and it was accessible at all times. The box was filled to capacity with 66 OTC naloxone kits. Staff took inventory of the boxes at least weekly using a paper log and restocked as needed. Over a six-month period, additional boxes were instituted at the WVU Crisis Support and Recovery Center (CSRC) in December 2023, BMC main entrance (March 2024), and the Jefferson Medical Center ED entrance (April 2024).

## Results

**Fig. 3:**
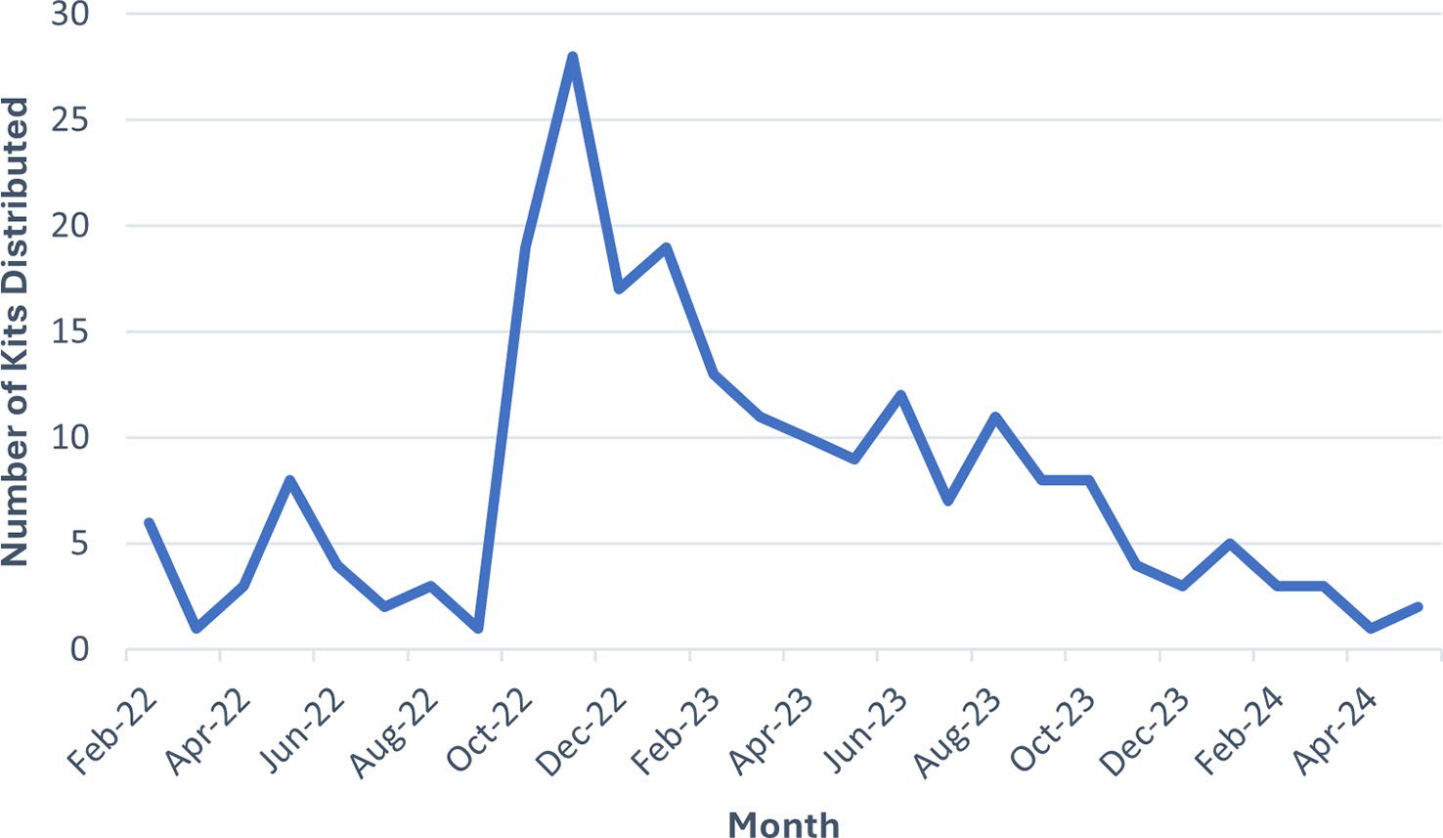
“Naloxone Kits Distributed Through “To-Go Meds” Per Month. Trend graph showing naloxone kits dispensed by physician orders in Berkeley Medical Center Emergency Department through “To-Go Meds” from program launch in February 2022 to May 2024

From December 2023-May 2024, **2**,**383** OTC naloxone kits (4,766 doses) were distributed from the four boxes placed at WVU Medicine East locations. On average, 397 kits were taken per month. By comparison, 17 kits were dispensed through physician orders in the emergency department via “To-Go Meds” over the same period, averaging approximately 3 kits per month. Since the start of the take-home naloxone program in February 2022, 221 kits were distributed by physician orders at BMC ED, with a peak of 28 kits in November 2022 (see Fig. 3). Increases in distribution by physician orders could be seen following word-of-mouth promotion of the program by Addictions Services staff, followed by decreases in distribution the following month. The promotional mousepads had little impact on physician ordering. Naloxone kit distribution increased slightly in January 2024, with five kits dispensed; however, this number declined to three in February and further decreased to a single kit in April, indicating a downward trend in utilization.

**Fig. 4:**
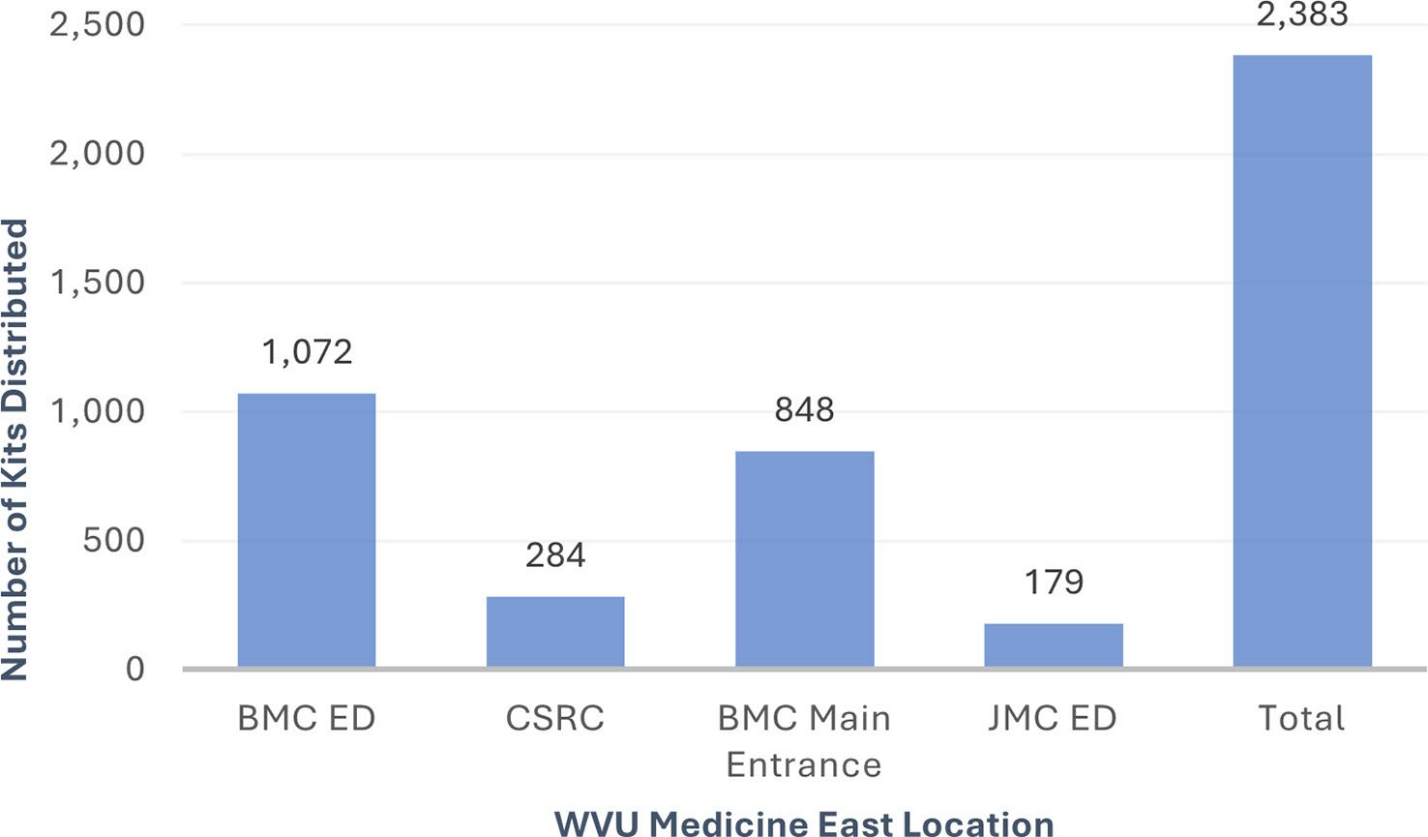
“Total Naloxone Kits Taken from Distribution Boxes by Location” Graph showing naloxone kits taken from distribution boxes by WVU Medicine Eastern Division location over a six-month period from December 2023-May 2024 (Berkeley Medical Center Emergency Department, Crisis Support & Recovery Center, Berkeley Medical Center Main Entrance, and Jefferson Medical Center Emergency Department)

**Fig. 5:**
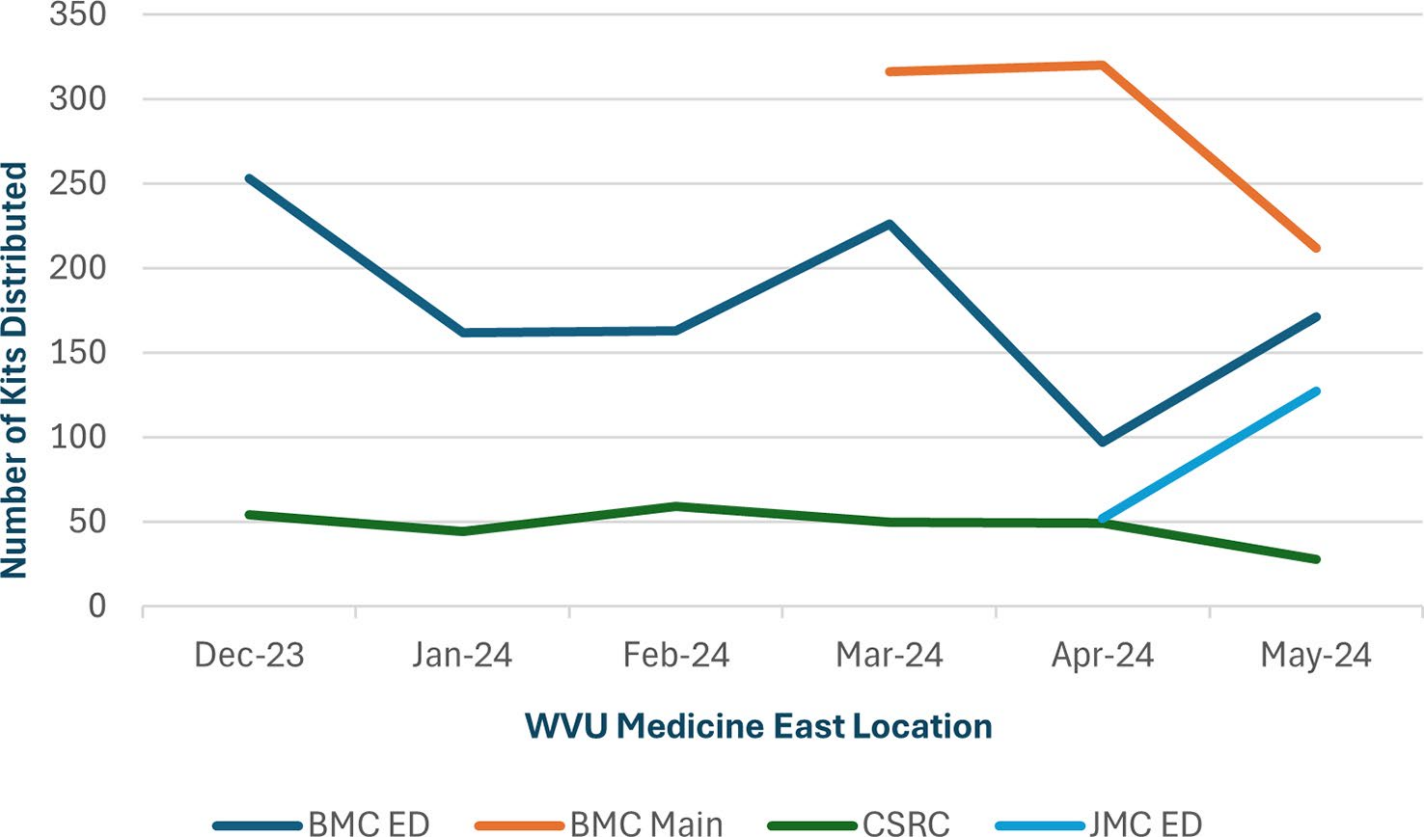
“Kits Taken from Distribution Boxes Per Month by Location” Trend graph showing naloxone kits taken from distribution boxes per month over the first 6-months of the project (December 2023-May 2024)

As shown in Fig. 4, the number of naloxone kits taken from the distribution boxes varied by location. Berkeley Medical Center experiences significantly higher patient traffic compared to the CSRC and JMC ED. Additionally, BMC ED was the first location to implement a distribution box, in December 2023. This was reflected by largest number of kits (1,702) being taken from the BMC ED box, followed by BMC Main entrance with 848 kits taken. Figure 5 shows the number of kits taken over time by location. The number of kits distributed per month remained relatively consistent, except for declines in April and May due to supply delays.

During the implementation period, Addiction Services staff noted that the naloxone distribution box program presented a potential opportunity for stigma reduction. Patient visits to the boxes allowed staff to provide education about naloxone, harm reduction, and opioid use disorder, with patients frequently initiating questions and conversations on their own. While staff occasionally engaged in conversations or witnessed individuals taking kits from the boxes, no personal information was recorded, preserving anonymity. Anecdotally, the staff noticed many adults 65 years of age and older accessing naloxone kits from the distribution boxes and inquiring about the medication.

## Lessons learned

Several barriers and facilitators for take-home naloxone programs were identified during the project. A barrier faced during implementation of both the “To-Go Meds” route and distribution boxes was gaining approval from the hospital system. Stigma may also pose a barrier, as hospital leadership could be concerned about community perception and whether the medical center’s logo, or that of community partners, appears on the naloxone distribution boxes. These barriers were overcome through relationship building and one-on-one discussions with ED physicians, pharmacists, and leadership roles. An additional barrier included supply delays from the state in April and May 2024 due to grant renewals, which caused the boxes to go empty on a few occasions. This barrier was addressed by placing a sign displaying a QR code linking to our local coalition website with additional locations and organizations that supply naloxone. The anonymity of the boxes, although beneficial, also meant we were unable to track demographics of the population receiving naloxone kits; thus, we relied solely on staff observations.

During implementation, a concern was raised that a single individual could take a disproportionate number of kits from the distribution boxes, skewing results and effectiveness. Staff monitored the supply of kits to identify whether large quantities were taken, particularly overnight. Some individuals were observed or informed staff that they were taking multiple kits to distribute to at-risk friends and family, but no instances of individuals taking an unreasonable number of kits were noted.

A key facilitator of the program was the ease of installing and using the distribution boxes once approved. Stocking the naloxone boxes and tracking distribution also requires very few steps. Strong community partnerships already existing through the local coalitions provided essential support and funding for the initiative. The program’s success was further strengthened by a dedicated team of public health professionals integrated into the healthcare system, driving and sustaining the initiative.

## Discussion

Implementation of naloxone distribution boxes significantly increased the quantity of naloxone provided to the community. The distribution boxes were popular throughout the six-month period, and a large number of kits were consistently taken each month. In contrast, the physician-ordered take-home naloxone program experienced fluctuations—increases during promotions by Addiction Services followed by periods of reduced momentum. Strategies such as “a non-interruptive alert” to increase ordering of take-home naloxone in emergency departments have been suggested in the literature and may be a beneficial addition to our program [21]. The results align with previous findings that passive, low-barrier naloxone distribution models such as vending machines and mail-based distribution can expand access to populations who might not otherwise receive naloxone through clinical settings [22, 23]. Our experience adds further support to growing evidence that convenience and anonymity are key facilitators in community naloxone uptake [24].

Patients 65 years and older receiving naloxone from the boxes was an unexpected benefit of the program, as we know older adults are increasingly at risk of opioid overdose [25]. Additionally, it’s possible that other populations were reached through the distribution boxes that would have been missed when naloxone is reserved for higher-risk patients in the ED. For example, take-home naloxone has been shown to prevent fatal overdose among children, who may be accidentally exposed to opioids in the home [26]. This finding contributes to a broader understanding of naloxone distribution impact, suggesting that expanding access through nontraditional channels can help reach underrepresented or overlooked populations, such as older adults and caregivers of young children. Such groups are rarely the focus of naloxone outreach efforts but may benefit from more intentional inclusion in future program planning.

Due to the large quantities of the medication released, the program has the potential to normalize naloxone as a first-aid measure within the community, similar to how other first-aid interventions, such as automated external defibrillators (AEDs) and EpiPens, have become more widely accepted through increased access [22, 27]. Integrating public health interventions into healthcare settings has been shown to enhance reach, improve health outcomes, and strengthen community-level prevention efforts [28].

Our area experienced a decline in overdose fatalities during the study period, and this trend continued afterward as the naloxone distribution boxes remained available. Although we cannot directly attribute this reduction to our program, particularly given the presence of other community resources, previous reports show that community-based naloxone availability has been shown to reduce overdose mortality at the population level [18, 19]. To our knowledge, our program has distributed the largest quantity of naloxone in the region. Replicating this model and conducting further studies could help assess the strength of this correlation. Additionally, tracking the specific locations and types of naloxone supplies used in overdose reversals may provide more insight into community impact. Some efforts to enhance data collection are already underway. One example is the Cryptids Campaign Pilot Program, a project of the WV Regional Drug Control Coordinators. This initiative features familiar images from Appalachian folklore, such as Bigfoot, displayed on the sides of naloxone boxes. Each box includes a QR code that links to a confidential, five-question survey designed to collect overdose data and also connects individuals to free naloxone and additional resources [29].

Variation in local infrastructure, community engagement, and pre-existing harm reduction efforts must be considered when applying this model to other regions. Therefore, generalizability may be limited to areas with similar organizational partnerships, healthcare system involvement, and willingness to support harm reduction interventions. Pilot testing in diverse settings will be important for assessing adaptability.

WVU Medicine Eastern Division hopes to continue to expand the take-home naloxone program systemwide. Plans are in place to add boxes at WVU Medicine Urgent Care Centers. Addiction Services has also consulted with Berkeley County Day Report and Berkeley County Recovery Resource Center to start their own naloxone distribution box programs and is actively working with hospital systems in Virginia, West Virginia, and Maryland through the Tri-State Collaborative to build additional programs. This cross-sector collaboration may serve as a model for other rural or underserved areas aiming to scale similar interventions. It demonstrates how partnerships between hospital systems, public health agencies, and community organizations can facilitate successful implementation and sustainability.

Although distribution rates from physician-ordered take-home naloxone were low, it still proved to be a valuable tool in providing high-risk patients with overdose prevention. Several physicians continue to use the “To-Go Meds” option, especially for overdose patients or other patients at risk of leaving against medical advice who would benefit from having medication in hand. This option eliminates the need for patients to remember to visit a distribution box before leaving the hospital. Additionally, Addiction Services relied on this take-home naloxone program during OTC supply delays. While the physician-distributed route did not yield the same volume as community boxes, it highlights the importance of maintaining multiple access points within a comprehensive naloxone strategy. These findings support literature indicating that redundancy in naloxone access pathways, clinical and non-clinical, is key to maximizing reach and meeting patients where they are [30].

As naloxone and opioid reversal medications become more normalized and accepted as a first-aid measure, further research is needed to identify additional locations to stock naloxone within the community, which may include schools, libraries, and other public buildings. With the addition of naloxone distribution boxes to the take-home naloxone program, we hope to see overdose fatality rates continue to decrease through 2024 and onward. Overall, our findings add to a growing body of literature supporting broad naloxone access and provide actionable insights for communities seeking to implement or expand similar efforts. Future research should examine long-term outcomes, cost-effectiveness, and the perspectives of people who use these resources to better understand implementation barriers and facilitators across diverse settings.

## Conclusion

Naloxone distribution boxes are an efficient means of offering vast quantities of life-saving medication to the community, alongside added benefits including anonymity and around-the-clock accessibility. Physician ordered take-home naloxone initiatives can be challenging to sustain over time but may offer important benefits, particularly for at-risk patients in the emergency department, and can serve as a valuable complement to naloxone distribution box programs.

## Data Availability

The datasets used and/or analyzed during the current study are available from the corresponding author on reasonable request.

## Abbreviations

BMC: Berkeley Medical Center
JMC: Jefferson Medical Center
CSRC: Crisis Support & Recovery Center
ED: Emergency department
MOUD: Medication for opioid use disorder
OTC: Over the counter
EMR: Electronic Medical Record
EMS: Emergency Medical Services
SUD: Substance use disorder
QRT: Quick Response Team
